# EHMTI-0236. A qualitative study of the functional impact of symptoms on migraine patients

**DOI:** 10.1186/1129-2377-15-S1-D53

**Published:** 2014-09-18

**Authors:** A Hareendran, S Mannix, A Skalicky, K Widnell, P Corey-Lisle, S Sapra

**Affiliations:** 1 Outcomes Research, Evidera, London, UK; 2 Outcomes Research, Evidera, Bethesda, USA; 3 Outcomes Research, Evidera, Seattle, USA; 4 Global Development, Amgen Inc., Thousand Oaks, USA; 5 Global Health Economics, Amgen Inc., Thousand Oaks, USA

## Introduction

Migraines have significant impacts on patients, resulting in functional and quality of life impairments. There is limited qualitative data that describe the functional limitations related to episodic (EM) and chronic migraines (CM).

## Aims

A qualitative study was conducted to understand migraine patients' experiences.

## Methods

Five U.S. clinical sites recruited patients to complete interviews with trained researchers. Subjects provided IRB-approved informed consent and met the following eligibility criteria: 18-60 years of age, and migraine history meeting IHS criteria. Interviews were conducted to elicit concepts that describe impacts on daily functioning. Transcripts were analyzed using qualitative methods.

## Results

21 EM and 11 CM patients were interviewed. Patient reported concepts were grouped into the following areas: physical function, everyday activities, social/relationship/leisure activities, and emotional responses. Physical function impacts included difficulty with moving head and bending, needing to lie down, limited movement, avoiding bright lights and loud noises; everyday activities included household chores, running errands, activities requiring concentration, interacting with others, performing tasks related to work and/or school. Social/relationship/leisure activity and emotional impacts included inability to make plans, avoiding people, feeling frustrated, moody, and guilty. Physical function was described as the most immediate impact from symptoms, with emotional and social impacts occurring during and after symptoms.

## Conclusion

Functional impacts from migraine symptoms described by EM and CM patients can be grouped into 3 domains: physical, emotional, and social. Physical function limitations play a role in the immediate migraine experience. Emotional and social impacts are experienced during and after migraine episodes.

